# Mechanisms and therapeutic implications of galectins regulating Epstein–Barr virus infection

**DOI:** 10.1016/j.virusres.2026.199768

**Published:** 2026-06-19

**Authors:** Jie-Yu Huang, Albright Dew Baua, Zih-Syuan Yang, Ching-I Tsui, Pin-Chen Chen, Wen-Hung Wang, Wanchai Assavalapsakul, Arunee Thitithanyanont, Yen-Hsu Chen, Sheng-Fan Wang

**Affiliations:** a Center for Tropical Medicine and Infectious Disease Research, Kaohsiung Medical University, Kaohsiung, 807378, Taiwan; b Department of Medical Laboratory Science and Biotechnology, Kaohsiung Medical University, Kaohsiung, 807378, Taiwan; c Department of Biotechnology, Kaohsiung Medical University, Kaohsiung, 807378, Taiwan; d School of Medicine, College of Medicine, National Sun Yat-Sen University, Kaohsiung, 804, Kaohsiung, Taiwan; e Department of Microbiology, Faculty of Science, Chulalongkorn University, Bangkok, 10330, Thailand; f Department of Microbiology, Faculty of Science, Mahidol University, Bangkok 10400, Thailand; g Division of Infectious Disease, Department of Internal Medicine, Kaohsiung Medical University Hospital, Kaohsiung, 807, Taiwan; h MSc. Program in Tropical Medicine, College of Medicine, Kaohsiung Medical University, Kaohsiung, 807378, Taiwan; i Department of Medical Research, Kaohsiung Medical University Hospital, Kaohsiung, 807, Taiwan

**Keywords:** Epstein–Barr virus, Galectins, Glycan–lectin interactions

## Abstract

Epstein–Barr virus (EBV) is a ubiquitous herpesvirus associated with a broad spectrum of malignancies and immune-mediated disorders, and growing evidence highlights the importance of host glycan–lectin interactions in shaping viral persistence and immune escape. Among these, galectins have emerged as key regulators of the EBV life cycle, influencing viral attachment, latency maintenance, lytic reactivation, and the remodeling of the tumor microenvironment. Galectin-1, -3, and -9 exhibit context-dependent functions that collectively modulate oncogenic signaling pathways, T‑cell exhaustion, regulatory T‑cell expansion, and innate immune sensing. Recent clinical studies further suggest that circulating galectins and galectin-enriched exosomes may serve as non-invasive biomarkers for disease progression and prognosis in EBV-associated malignancies. Despite these advances, major knowledge gaps remain regarding member-specific functions, compensatory galectin networks, and the spatiotemporal dynamics of galectin regulation during infection. Targeting the galectin–glycan axis therefore represents a promising frontier for host-directed antiviral and anticancer therapies, with the potential to disrupt viral latency, restore antiviral immunity, and improve clinical outcomes in EBV-driven diseases.

## Highlights

• Galectin‑1, ‑3, and ‑9 as major modulators of EBV infection.
• Galectin‑mediated modulation of viral latency, immune evasion, and oncogenesis.
• Dual immunological roles of galectins in T‑cell exhaustion and antiviral responses.
• Therapeutic potential of targeting the galectin–glycan axis in EBV‑associated diseases.

## 1 Introduction

Epstein-Barr virus (EBV), also known as human herpesvirus 4 (HHV-4), is a highly prevalent double-stranded DNA virus that infects over 95% of the global adult population (Damania et al., 2022; Indari et al., 2024). Following primary infection—often asymptomatic or manifesting as infectious mononucleosis—EBV establishes lifelong latency in memory B cells. While typically controlled by host immunity, EBV persistence is associated with a spectrum of malignancies, including Hodgkin lymphoma, nasopharyngeal carcinoma, and EBV-positive gastric carcinoma (Huang et al., 2023; Hatton et al., 2014). Despite its oncogenic potential and global disease burden, there are currently no licensed antiviral therapies specifically approved for EBV-related diseases (Soldan et al., 2022; Chakravorty et al., 2022).

To persist in the host and avoid immune elimination, EBV employs a range of immune evasion strategies. These include downregulation of major histocompatibility complex (MHC) molecules, secretion of viral interleukin-10 (vIL-10), induction of regulatory T cells (Tregs), and exploitation of immune checkpoints such as PD-1 and TIM-3 (Silva et al., 2024; Bauer et al., 2021). While viral mechanisms of immune escape are well characterized, increasing attention has turned to the role of host-derived glycan–lectin interactions in shaping EBV pathogenesis. Lectins are a broad class of glycan-recognizing proteins that influence numerous cellular processes, including signal transduction, cell–cell interaction, and immune regulation (Watanabe et al., 2019; Laubli and Varki, 2020; Rabinovich and Croci, 2012; Chettri et al., 2021; Sharon and Lis, 1989).

Among these, galectins—a unique family of β-galactoside-binding proteins—have emerged as key regulators in host–pathogen interactions, particularly during viral infections (Tung et al., 2025; Wang et al., 2020; Wang et al., 2024). Galectins are widely distributed across various tissues, especially in immune-related organs and cells. Each galectin is characterized by a carbohydrate recognition domain (CRD) of roughly 130 amino acids, which mediates specific binding to β-galactoside-containing glycoconjugates. To date, 15 distinct galectins have been identified in mammals (Vasta, 2012) (Troncoso et al., 2023). Although synthesized without a classical signal peptide, galectins are capable of being secreted through unconventional pathways, enabling them to exert functions both inside and outside the cell (Troncoso et al., 2023). Structurally, galectins are classified into three types: prototype (e.g., galectin-1, Gal-1), tandem-repeat (e.g., galectin-9, Gal-9), and chimera-type (e.g., galectin-3, Gal-3).

Importantly, galectins exert distinct biological functions depending on their subcellular localization. Intracellular galectins regulate processes such as signal transduction, vesicular trafficking, mitochondrial stability, and innate immune sensing, thereby influencing viral replication, latency, and cell survival. In contrast, galectins present in the extracellular milieu—most often released via unconventional secretion pathways—act as soluble lectins that bind glycosylated receptors on neighboring cells, modulating virus–cell attachment, immune cell activation, and intercellular communication (Tung et al., 2025; Wang et al., 2020).

This review consolidates current evidence on the involvement of galectins in EBV infection and pathogenesis. We focus on their regulatory roles in viral latency, immune modulation, and tumor progression. Furthermore, we explore the therapeutic implications of targeting galectin‑mediated pathways in EBV‑associated diseases and discuss their potential as diagnostic biomarkers and intervention points in host‑directed antiviral therapy.

## 2 Dual roles of galectins in the EBV life cycle: Facilitation and inhibition

**Fig. 1:**
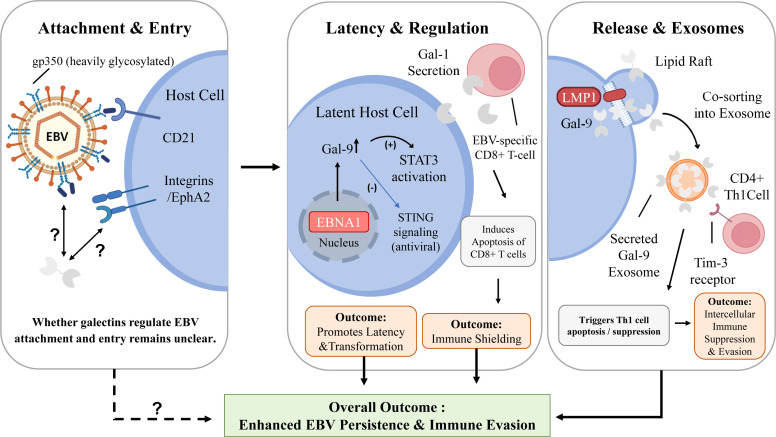
Roles of galectins across key stages of the Epstein–Barr virus (EBV) life cycle. This schematic illustrates how galectins modulate EBV infection. Left: The potential roles of galectins in regulating interactions between EBV glycoproteins and host entry receptors during viral attachment remain unresolved. Middle: In latently infected cells, Gal‑9 enhances STAT3 signaling while suppressing STING‑mediated antiviral responses. These intracellular effects may indirectly influence antiviral T‑cell immunity. Right: LMP1 and Gal‑9 co-localize within lipid raft–derived exosomes that engage TIM‑3 on Th1 (CD4⁺) T cells, promoting immunosuppression.

Through their capacity to bind β-galactoside-containing glycans to viral and host proteins, galectins are becoming more widely acknowledged as context-dependent host factors that influence several stages of the EBV life cycle. Galectins can either promote EBV persistence or, when inhibited, disrupt infection and restore antiviral immune pressure by affecting viral attachment, intracellular trafficking, replication–latency choices, immune evasion, and virion release. We have integrated the discussion of both facilitative and inhibitory roles here to provide a unified view of how galectins act as "double-edged swords" during viral persistence (Fig. 1).

### 2.1 Galectin in EBV attachment and entry

Glycoprotein–receptor interactions at the host cell surface are how EBV starts infection. The envelope glycoprotein gp350 binds to CD21 (CR2) to mediate viral attachment in B cells, while CD21-independent mechanisms involving interactions between the gH/gL complex and cellular integrins (e.g., αvβ6 and αvβ8) as well as alternative receptors like EphA2 and neuropilin-1 (NRP1) facilitate epithelial cell entry (Sathiyamoorthy et al., 2016; Hutt-Fletcher, 2015). Following attachment, the viral fusion protein gB executes membrane fusion (Hutt-Fletcher, 2015). Although EBV entry receptors are well characterized, the viral envelope is extensively decorated with N‑ and O‑linked glycans, forming a “glycan shield” that is theoretically accessible to host lectins (Kanekiyo et al., 2015; Serafini-Cessi et al., 1989; Szakonyi et al., 2006). Certain galectin members have been reported to interact with host receptors and influence the entry of some viruses (Tung et al., 2025; Wang et al., 2020; Wang et al., 2024); however, no experimental evidence to date supports a role for galectins in EBV attachment or entry.

### 2.2 Galectins in EBV latency maintenance and host antiviral signaling

The interplay between EBV and host cellular factors is central to determining whether the virus establishes latency or enters lytic replication (Shannon-Lowe et al., 2009; Yu and Robertson, 2023). Beyond their potential involvement in viral entry, galectins modulate intracellular signaling pathways that influence host cell survival, innate immune sensing, and the maintenance of viral episomes. Following infection of B cells or epithelial cells, EBV adopts a latent program characterized by restricted expression of EBNA1, LMP1, and LMP2, enabling long‑term persistence while evading immune detection (Shannon-Lowe et al., 2009; Yu and Robertson, 2023). During this phase, several intracellular galectins are upregulated, reflecting their roles in supporting the cellular environment required for viral persistence.

Among these lectins, Gal‑9 has emerged as an important regulator of EBV latency. Xu et al. demonstrated that EBNA1 drives Gal‑9 expression, and elevated Gal‑9 promotes B‑cell transformation by activating STAT3 and suppressing STING‑dependent antiviral signaling (Xu et al., 2023a). This Gal‑9–STAT3–STING axis represents a central mechanism through which EBV reinforces latency and protects infected cells from innate immune surveillance. Notably, genetic or antibody‑mediated inhibition of Gal‑9 restores cGAS–STING signaling and type I interferon production, thereby disrupting latent infection and limiting the outgrowth of EBV‑infected B cells (Xu et al., 2023b).

In parallel, Gal‑1 contributes to EBV persistence primarily through immune‑modulatory mechanisms. Gandhi et al. showed that Gal‑1 induces apoptosis and functional impairment of EBV‑specific CD8⁺ T cells in classic Hodgkin lymphoma, creating an immunosuppressive microenvironment that shields latently infected cells from cytotoxic clearance (Gandhi et al., 2007). Thus, while both Gal‑1 and Gal‑9 facilitate EBV persistence, they do so through distinct mechanisms—Gal‑9 by modulating intracellular antiviral signaling and Gal‑1 by suppressing antiviral T‑cell immunity.

Together, these findings highlight galectins as integral regulators of EBV latency, acting through complementary intracellular and immunological pathways that collectively sustain viral persistence.

### 2.3 Galectin‑mediated exosomal release and immune suppression

Emerging evidence suggests that galectins orchestrate the release of viral components to actively modulate the host immune microenvironment. The assembly of these suppressive vehicles begins at the plasma membrane. Providing molecular insight, Pioche-Durieu et al. revealed that in NPC cells, LMP1 physically interacts with Gal-9 within membrane lipid rafts, a localization that likely governs their subsequent co-sorting into exosomes (Pioche-Durieu et al., 2005).

Once released, these Gal‑9–containing exosomes exert potent immunosuppressive effects. Klibi et al. showed that exosomal Gal‑9 binds the TIM‑3 receptor on EBV‑specific CD4⁺ Th1 cells, triggering apoptosis and suppressing IFN‑γ production (Klibi et al., 2009). Importantly, the exosomal membrane protects Gal‑9 from proteolytic degradation, thereby prolonging its immunosuppressive activity and enabling systemic diffusion in the circulation (Klibi et al., 2009).

This mechanism appears conserved across EBV‑infected cell types. Xu et al. further reported that EBV‑infected B cells package Gal‑9 into exosomes, and Gal‑9–dependent suppression of STING signaling contributes to viral persistence (Xu et al., 2023a). Whether exosomal Gal‑9 directly modulates STING activity in neighboring cells remains to be experimentally clarified.

Collectively, these findings highlight galectin‑mediated exosomal release as a critical mechanism of EBV‑driven immune evasion, suggesting that disruption of galectin sorting or exosome biogenesis may represent a promising therapeutic strategy.

## 3 Implications of galectins as pathogenic factors in EBV-associated oncogenesis

EBV contributes to cancer development through multiple mechanisms, particularly during latent infection. Latency-associated proteins such as LMP1 and EBNA1 activate host oncogenic signaling pathways (e.g., NF-κB, PI3K/AKT), disrupt immune regulation, and promote cell survival, proliferation, and migration (Cheerathodi and Meckes, 2018; Lo et al., 2021). In addition to direct viral effects, EBV-associated malignancies—such as Hodgkin lymphoma, nasopharyngeal carcinoma (NPC), and gastric carcinoma—are also characterized by chronic inflammation, immune evasion, and a tumor-permissive microenvironment (Bauer et al., 2021; Li et al., 2025). Within this context, galectins have emerged as important modulators rather than passive bystanders.

Mechanistically, several EBV gene products contribute to the dysregulation of galectin expression. Ouyang et al. demonstrated that in EBV⁺ PTLD, LMP1 induces Gal-1 expression through AP-1 activation (Ouyang et al., 2011). LMP1 signaling has also been associated with increased Gal-9 expression and its incorporation into exosomes (Pioche-Durieu et al., 2005; Klibi et al., 2009), although the functional consequences of this regulation remain incompletely defined.

In nasopharyngeal carcinoma (NPC), Li et al. reported that Gal-3 is upregulated and promotes tumor cell proliferation through ERK1/2 and Akt signaling (Li et al., 2021). Klibi et al. further showed that EBV‑infected epithelial cells secrete Gal‑9–containing exosomes, and these exosomes can induce apoptosis in TIM‑3⁺ CD4⁺ T cells in vitro (Klibi et al., 2009). In contrast, Gal-1 plays a more clearly defined role in EBV-positive Hodgkin lymphoma, where it contributes to immune escape by impairing EBV-specific CD8⁺ T-cell responses (Gandhi et al., 2007). Collectively, these findings indicate that galectins participate in EBV-associated oncogenesis through context-dependent and cancer-specific mechanisms, with Gal-1, Gal-3, and Gal-9 acting through distinct pathways in lymphoid versus epithelial malignancies.

### 3.1 Galectins in EBV‑associated lymphomas

Beyond nasopharyngeal carcinoma, galectins play important roles in certain EBV-associated lymphoproliferative diseases. Gal-1 is particularly well characterized in classic Hodgkin lymphoma (cHL) (Gandhi et al., 2007; Rodig et al., 2008). Reed–Sternberg cells express high levels of Gal-1, which contributes to immune evasion by inducing apoptosis and functional impairment of EBV-specific CD8⁺ T cells, thereby establishing a profoundly immunosuppressive tumor microenvironment (Gandhi et al., 2007; Rodig et al., 2008). These findings are consistent with broader evidence that Gal-1 suppresses Th1 and cytotoxic responses in hematologic malignancies (Rubinstein et al., 2004).

In EBV-positive diffuse large B-cell lymphoma (DLBCL) and post-transplant lymphoproliferative disorder (PTLD), Gal-9 is frequently upregulated. Xu et al. demonstrated that EBNA1 induces Gal-9 expression, which in turn suppresses STING-mediated antiviral signaling and promotes the outgrowth of EBV-infected B cells (Xu et al., 2023b). Additional studies have shown that Gal-9–containing exosomes can induce apoptosis of TIM-3⁺ T cells, contributing to immune suppression in EBV-associated lymphomas (Klibi et al., 2009). Moreover, LMP1-driven Gal-1 upregulation has been documented in EBV-positive PTLD tissues (Ouyang et al., 2011), further supporting the involvement of multiple galectin family members in lymphomagenesis.

Together, these findings indicate that Gal-1 and Gal-9 regulate EBV-associated lymphomas through distinct but complementary mechanisms, including T-cell suppression, modulation of innate antiviral signaling, and remodeling of the tumor microenvironment. These lymphoma-specific pathways differ substantially from those observed in epithelial malignancies such as NPC, underscoring the need to consider galectin biology within the context of each EBV-associated disease.

Emerging evidence also suggests potential translational relevance of galectin dysregulation in EBV‑associated lymphomas. Elevated Gal‑1 and Gal‑9 expression has been explored as a basis for biomarker development, including Gal‑1 upregulation in EBV‑positive lymphoproliferative lesions (Ouyang et al., 2011) and the detection of circulating Gal‑9–enriched exosomes in patients with EBV‑associated malignancies (Klibi et al., 2009). From a therapeutic perspective, preclinical studies indicate that targeting Gal‑9 or the Gal‑9/TIM‑3 axis may modulate immune suppression or restore antiviral signaling, as demonstrated by Gal‑9 inhibition reactivating STING‑dependent pathways (Xu et al., 2023a) and TIM‑3 blockade alleviating T‑cell exhaustion (Kandel et al., 2021). Although these strategies have not yet been evaluated specifically in EBV‑associated lymphomas, they highlight the broader therapeutic potential of galectin‑directed interventions in this disease context.

## 4 Immunoregulation by galectins during EBV infection

**Fig. 2:**
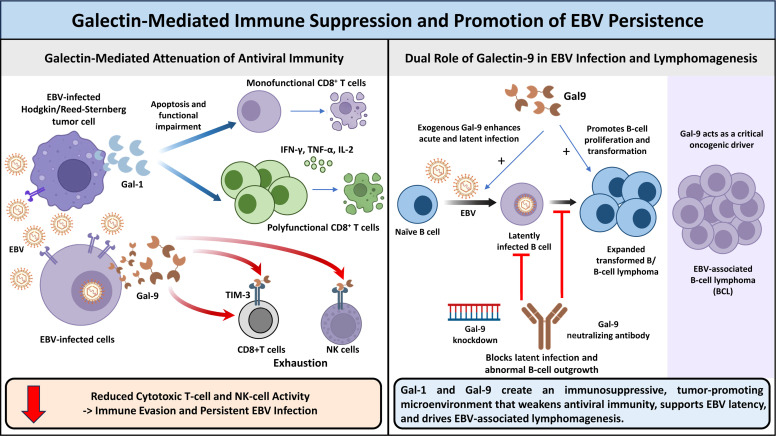
Galectin‑mediated immunoregulation during EBV infection. This figure illustrates the dual roles of galectins in shaping antiviral immunity. Left: Gal‑1 and Gal‑9 promote an immunosuppressive environment by inducing apoptosis or functional exhaustion of EBV‑specific T cells and NK cells. Right: Gal‑9 supports EBV latency and B‑cell outgrowth through modulation of intracellular antiviral signaling pathways.

The interaction between EBV and the host immune system is defined by a continuous tug-of-war. While the host deploys natural killer (NK) cells, type I interferons, and cytotoxic T cells to curb viral replication, EBV counters these defenses through sophisticated immunomodulatory tactics. In this complex battlefield, galectins have surfaced as versatile mediators, capable of tipping the scales toward either immune suppression or activation (Fig. 2).

### 4.1 Attenuation of immune responses and facilitation of EBV persistence

To secure its persistence, EBV actively exploits galectins to engineer an immunosuppressive niche. In EBV‑positive Hodgkin lymphoma, Gandhi et al. demonstrated that elevated Gal‑1 suppresses cytotoxic T‑cell activity by inducing apoptosis and functional impairment of EBV‑specific CD8⁺ T cells (Gandhi et al., 2007). Kandel et al. further identified a parallel mechanism of exhaustion, showing that Gal-9 engagement with the TIM-3 receptor renders both T cells and natural killer (NK) cells functionally impaired (Kandel et al., 2021), although the strength and directness of this interaction remain under active debate.

Nevertheless, this viral strategy is not absolute. Smith et al. reported a “quality‑dependent” resistance, showing that polyfunctional EBV‑specific CD8⁺ T cells—those capable of producing multiple cytokines—are more resistant to Gal‑1‑mediated apoptosis than monofunctional T cells (Smith et al., 2009). This suggests that while EBV co‑opts galectins to dampen immunity, a robust, high‑quality T‑cell response may partially overcome this blockade. Clinical evidence also supports Gal‑9 dysregulation during acute EBV infection. In children with infectious mononucleosis, Xu et al. reported elevated plasma Gal‑9 and TIM‑3 expression, accompanied by increased IL‑17A and IL‑22 levels, suggesting activation of an immunoregulatory axis that may contribute to T‑cell dysfunction during primary EBV infection (Xu et al., 2023b).

### 4.2 Modulation of immune response: The dual role of Gal-9 and challenges in inhibiting EBV replication

Throughout the progression of EBV infection, Gal-9 exhibits paradoxically multifaceted properties. Although several galectin family members have been reported to exert antiviral effects in other viral systems, EBV‑specific evidence indicates a distinct role for Gal‑9. Recent mechanistic studies highlight Gal‑9 as a critical facilitator of latent infection and lymphomagenesis. Xu et al. demonstrated that knocking down or blocking Gal-9 expression effectively inhibits the establishment of latent infection and the abnormal outgrowth of EBV-infected B cells. Conversely, the administration of exogenous Gal-9 protein during the early stages of infection significantly promotes both acute and latent EBV infection, accelerating the proliferation and transformation of infected B cells (Xu et al., 2023a). These findings suggest that Gal‑9 contributes to EBV‑associated B‑cell lymphomagenesis by supporting latent infection and B‑cell outgrowth, rather than acting merely as an immune‑modulatory molecule.

## 5 Therapeutic strategies and clinical translation challenges

The identification of galectins as key regulators of EBV persistence and oncogenesis highlights their potential as therapeutic targets. By disrupting galectin‑mediated immunosuppression and restoring intracellular antiviral signaling, pharmacological intervention offers a promising avenue for EBV‑associated malignancies. However, successful translation requires strategies that integrate direct inhibition with rational combinatorial approaches. This section summarizes emerging therapeutic directions while acknowledging the translational challenges posed by the functional redundancy of the galectin family.

### 5.1 Galectin inhibitors and immunotherapy synergy

Recent mechanistic studies demonstrate that galectin blockade can reverse critical pathways supporting EBV persistence. Gal‑9 knockdown, for example, not only diminishes its immunosuppressive activity but also reactivates the cGAS–STING antiviral pathway, restoring type I interferon production essential for controlling latent infection (Xu et al., 2023b). Gal‑1 inhibition may similarly enhance EBV‑specific T‑cell responses by reducing Gal‑1–mediated suppression, particularly in functionally competent, polyfunctional CD8⁺ T cells that exhibit greater resistance to Gal‑1–induced dysfunction (Smith et al., 2009). These effects reflect broader immunoregulatory mechanisms rather than EBV‑specific Treg biology, which remains insufficiently defined.

**Fig. 3:**
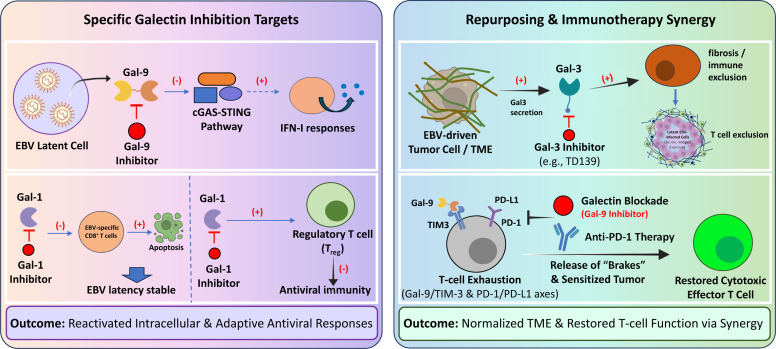
Therapeutic strategies targeting galectins in EBV‑associated diseases. This schematic highlights potential intervention points within galectin‑regulated pathways. Upper left: Gal‑9 inhibition restores cGAS–STING–mediated type I interferon responses. Lower left: Gal‑1 blockade reduces T‑cell suppression and may enhance antiviral immunity. Upper right: Gal‑3 inhibition modulates stromal and microenvironmental factors. Lower right: Combined galectin blockade with PD‑1/PD‑L1 inhibition may improve T‑cell effector function.

Repurposing clinical‑stage inhibitors provides an accelerated translational route. Gal‑3 inhibitors such as TD139 (GB0139), currently evaluated in fibrotic and inflammatory lung disease (Slack et al., 2021; Humphries et al., 2022), may hold relevance for EBV‑driven tumors by modulating stromal fibrosis and altering the tumor microenvironment. Although EBV‑specific evidence is lacking, such agents may serve as microenvironment‑modifying “priming therapies.” Galectin inhibition may also potentiate immune checkpoint blockade (ICB). Because the Gal‑9/TIM‑3 axis is implicated as a parallel inhibitory pathway to PD‑1/PD‑L1 (Kandel et al., 2021; Okoye et al., 2020; Sakuishi et al., 2011), even though the direct biochemical interaction between Gal‑9 and TIM‑3 remains uncertain, co‑blockade strategies may still relieve additional layers of T‑cell exhaustion and enhance responsiveness to anti‑PD‑1 therapy (Fig. 3).

A summary of current pharmacological strategies and their validated biological effects in EBV‑related models is provided in Table 1.

**Table 1:** Pharmacological targeting of galectins in EBV infection and associated malignancies: Strategies, mechanisms, and outcomes.

Galectin	Disease Context / Model	Targeting Strategy / Pharmacological Process	Mechanism of Action	Observed Biological Effect	Ref.
Gal-1	EBV-positive Hodgkin Lymphoma	Genetic Silencing (siRNA) / Antibody Blockade	-Inhibition of Gal-1 mediated apoptosis in effector T cells	-Suppresses CTL apoptosis and restores antiviral immune pressure in the tumor microenvironment	(Gandhi et al., 2007)
Gal-3	Nasopharyngeal Carcinoma (NPC)	Targeting Oncogenic Signaling (siRNA)	Inhibition of Gal-3 mediated ERK1/2 and Akt activation	Suppresses tumor cell proliferation and migration	(Li et al., 2021)
Gal-9	EBV-transformed B-cell Lymphomas	Genetic Deficiency / Inhibition (shRNA)	Reactivation of the cGAS-STING DNA sensing pathway	Enhances type I interferon (IFN) production and limits viral latency maintenance	(Xu et al., 2023)
	Nasopharyngeal Carcinoma (NPC) / Solid Tumors	Antibody Blockade (Anti-TIM-3 / Anti-Gal-9)	Inhibition of the TIM-3 co-inhibitory signaling pathway	Reverses T-cell exhaustion and restores cytotoxic effector function	(Okoye et al., 2020)
	Nasopharyngeal Carcinoma (NPC)	Neutralization of Exosomal Gal-9	Prevention of exosome uptake and downstream signaling	Restores EBV-specific CD4+ T cell survival in the NPC microenvironment	(Klibi et al., 2009; Keryer-Bibens et al., 2006)

Footnote : This table summarizes key therapeutic strategies evaluated in preclinical and clinical models, highlighting the mechanism of action and biological outcomes of targeting Galectin-1, −3, and −9.

*Abbreviations*: siRNA, small interfering RNA; shRNA, short hairpin RNA; CTL, cytotoxic T lymphocyte; Treg, regulatory T cell; NPC, nasopharyngeal carcinoma.

### 5.2 Barriers to translation

Despite encouraging preclinical evidence, clinical translation is complicated by the substantial functional redundancy within the galectin family. Gal‑1, ‑3, and ‑9 frequently regulate overlapping processes—including apoptosis, immune suppression, and stromal remodeling—raising the possibility that inhibition of a single galectin may be insufficient. A key unresolved question is whether blockade of one galectin (e.g., Gal‑9) triggers compensatory upregulation of others (e.g., Gal‑3), thereby sustaining the immunosuppressive phenotype. This adaptive “galectin network” response remains poorly characterized in EBV‑infected systems and represents a critical knowledge gap for therapeutic development.

## 6 Galectins as potential biomarkers in EBV-associated malignancies

There is an urgent clinical need for accurate, non-invasive biomarkers to refine the diagnosis, prognosis, and therapeutic monitoring of EBV-associated diseases. While the quantification of EBV DNA in blood or tumor tissue remains the diagnostic gold standard, it primarily reflects viral load and often fails to capture the complexity of the host immune response or the tumor microenvironment. Galectins, given their secretion into the extracellular space and circulation, are increasingly recognized as potential candidates for "liquid biopsy" applications.

Recent clinical investigations have begun to validate the utility of circulating galectins as diagnostic indicators. Moving beyond mechanistic descriptions, Klibi et al. and Keryer-Bibens et al. demonstrated the feasibility of detecting Gal-9-enriched exosomes in the plasma of patients with nasopharyngeal carcinoma, supporting an association between tumor presence and circulating Gal-9–positive exosomes (Klibi et al., 2009; Keryer-Bibens et al., 2006). Similarly, in the context of lymphoproliferative disorders, Ouyang et al. identified significantly elevated Gal-1 expression in EBV-positive tissues, suggesting that Gal-1 expression may aid in distinguishing EBV-positive lymphoproliferative lesions (Ouyang et al., 2011).

Beyond diagnosis, galectin levels may also serve as critical prognostic tools for risk stratification. Specifically regarding EBV-driven malignancies, Chen et al. identified that in patients with recurrent nasopharyngeal carcinoma, the co-expression of Gal-9 and TIM-3 correlates strongly with poor patient survival (Chen et al., 2017). This clinical correlation likely reflects, at least in part, the severity of EBV-induced immune exhaustion, where the virus exploits these pathways to evade surveillance even in late-stage disease. Collectively, these findings highlight the translational value of galectins: measuring their levels could complement traditional virological and histopathological assessments, providing a more comprehensive snapshot of the disease state to guide clinical decision-making.

## 7 Discussion

Galectins have emerged as central regulators at the intersection of EBV biology, host immunity, and tumor microenvironmental remodeling. By integrating mechanistic, immunological, and clinical evidence, this review highlights how Gal-1, Gal‑3, and Gal‑9 collectively shape the trajectory of EBV infection and EBV‑associated malignancies, particularly nasopharyngeal carcinoma (NPC). These lectins influence multiple stages of disease—from viral persistence and immune evasion to stromal remodeling and therapeutic resistance—positioning the galectin–glycan axis as a promising but still underexplored target for intervention (Silva et al., 2024; Bauer et al., 2021; Tung et al., 2025; Wang et al., 2020; Gandhi et al., 2007; Klibi et al., 2009; Cheerathodi and Meckes, 2018; Lo et al., 2021).

Despite substantial progress, several conceptual gaps limit the translation of galectin biology into clinical applications. One major challenge is the incomplete understanding of the spatiotemporal dynamics of galectin expression during the EBV life cycle. EBV latency and lytic reactivation involve rapid, context‑dependent shifts in viral gene expression and immune recognition (Damania et al., 2022; Indari et al., 2024; Shannon-Lowe et al., 2009; Yu and Robertson, 2023), yet most existing studies rely on static *in vitro* models that cannot capture corresponding changes in galectin secretion, intracellular trafficking, or receptor engagement (Banfer et al., 2018; Zhang et al., 2025; Liu and Stowell, 2023; Sehrawat et al., 2010). Because these transitions are tightly linked to immune detection and viral dissemination, defining the temporal “windows of vulnerability” in galectin regulation will be essential for identifying when galectin‑directed therapies may exert maximal antiviral or immunomodulatory benefit.

A second challenge lies in the functional redundancy and compensatory interactions among Gal‑1, Gal‑3, and Gal‑9. These galectins converge on overlapping immunosuppressive pathways—including T‑cell exhaustion, regulatory T‑cell expansion, and myeloid polarization (Tung et al., 2025; Wang et al., 2020; Wang et al., 2024; Vasta, 2012; Kandel et al., 2021; Okoye et al., 2020)—suggesting that EBV‑associated tumors may rely on a broader “galectin network” rather than a single dominant lectin to maintain immune escape. This redundancy raises the possibility that monotherapy targeting a single galectin may be insufficient. Comprehensive mapping of this network through integrated transcriptomic, glycomic, and secretomic approaches will be critical for identifying dominant regulatory nodes and designing rational combination strategies.

The dual intracellular and extracellular functions of galectins further complicate therapeutic development. Extracellular Gal‑9 blockade may reinvigorate exhausted T cells by disrupting TIM‑3–mediated inhibitory signaling (Kandel et al., 2021; Okoye et al., 2020; Chen et al., 2017), whereas intracellular galectin functions—supported by emerging evidence of diverse galectin–protein interactions (Vasta, 2012; Troncoso et al., 2023) may influence innate immune pathways relevant to EBV latency. Similarly, Gal‑3 contributes to stromal fibrosis and tumor cell survival (Li et al., 2021; Slack et al., 2021; Humphries et al., 2022), while Gal‑1 modulates T‑cell apoptosis and antigen‑specific immune suppression (Gandhi et al., 2007; Ouyang et al., 2011; Smith et al., 2009). These compartment‑specific roles imply that precision targeting—rather than broad lectin inhibition—will be necessary to achieve therapeutic efficacy while minimizing off‑target effects.

In addition, although early studies suggested that Gal-9 may suppress Th1 immunity through engagement of TIM-3, recent structural and biophysical analyses have questioned whether Gal-9 directly binds TIM-3 with high affinity (Leitner et al., 2013; Sabatos-Peyton et al., 2018). High-resolution structural studies of the TIM-3 IgV domain demonstrated ligand specificity for phosphatidylserine and CEACAM1 rather than β-galactoside–containing glycans, and surface plasmon resonance assays failed to detect strong Gal-9–TIM-3 interactions (Leitner et al., 2013; Sabatos-Peyton et al., 2018).These findings do not exclude a potential role for Gal-9 in shaping the immune microenvironment of EBV-associated tumors, but they indicate that some Gal-9–mediated effects may occur through TIM-3–independent pathways. This emerging uncertainty underscores the need for cautious interpretation of Gal-9–TIM-3 signaling in epithelial malignancies such as NPC and highlights the importance of further mechanistic studies to clarify the receptor landscape of Gal-9 in EBV-infected tissues (Leitner et al., 2013; Sabatos-Peyton et al., 2018; Anderson et al., 2016).

Although no galectin‑directed therapies are currently approved for EBV‑associated cancers, several emerging opportunities are noteworthy. Gal‑3 inhibitors such as TD139 (GB0139), already in clinical trials for fibrotic lung disease, may serve as priming agents by reducing stromal fibrosis and improving immune infiltration in EBV‑driven tumors (Slack et al., 2021; Humphries et al., 2022). However, the high structural homology among galectin carbohydrate‑recognition domains presents a significant specificity challenge. This underscores the need for allosteric inhibitors, multivalent glycomimetics, or biologics capable of selectively modulating pathogenic galectin functions without broadly disrupting physiological glycan–lectin interactions.

Ultimately, galectin inhibition is unlikely to be curative as monotherapy. The most promising applications lie in synergistic combinations, particularly co‑targeting Gal‑9 or Gal‑3 alongside PD‑1/PD‑L1 blockade to overcome entrenched immune exhaustion (Kandel et al., 2021; Okoye et al., 2020; Chen et al., 2017). Progress in this area will depend on the development of physiologically relevant models that recapitulate the galectin network within EBV‑infected tissues and on the integration of companion diagnostics, such as circulating galectin levels or galectin‑rich exosomes, to guide patient selection and monitor therapeutic response.

Taken together, the evidence positions galectins as integral components of the host–virus interface in EBV‑associated malignancies. Rather than acting as isolated effectors, Gal‑1, Gal‑3, and Gal‑9 form an interconnected immunoregulatory network that EBV exploits to sustain latency, remodel the tumor microenvironment, and evade immune surveillance. Advancing galectin‑directed therapies will require a systems‑level understanding of how these lectins interact with viral programs, immune checkpoints, and stromal elements across disease stages. By integrating mechanistic insights with emerging clinical observations, future research can define how best to leverage galectin inhibition—alone or in combination with immunotherapy—to disrupt EBV persistence and improve outcomes for patients with EBV‑driven cancers.

## 8 Conclusion

Galectins are multifunctional regulators of the EBV life cycle, influencing viral persistence, immune modulation, and tumor progression. Among them, Gal‑1, Gal‑3, and Gal‑9 play context‑dependent roles that can either reinforce EBV‑driven oncogenesis or, when inhibited, restore antiviral immunity and remodel the tumor microenvironment. These dual functions highlight the therapeutic potential of targeting the galectin–glycan axis in EBV‑associated malignancies. Moving forward, three priorities will be essential. First, clarifying the spatiotemporal dynamics of galectin regulation during EBV latency and reactivation will help define optimal therapeutic windows. Second, mapping the interconnected galectin network will guide rational combination strategies that address functional redundancy. Third, the development of compartment‑specific inhibitors and exosome‑directed approaches may enable more precise modulation of galectin activity within EBV‑infected tissues. Together, these insights position galectin‑focused interventions as a promising avenue for host‑directed antiviral and anticancer therapy. Strategic modulation of galectin pathways has the potential to disrupt EBV latency, recondition the tumor microenvironment, and enhance the efficacy of existing immunotherapies in EBV‑associated diseases.

## Funding

This work was supported by grants from the National Science and Technology Council, R.O.C. (112-2320-B-037-032-MY3 & 113-2740-M-037-001), the Kaohsiung Medical University Research Center (KMU-TC113B01), the Kaohsiung Medical University Research Foundation (KMU-M112007), the NYCU-KMU Joint Research Project (NYCU-KMU-113-I004), the NSYSU-KMU Joint Research Project (#NSYSUKMU 113-I04), and a grant from the Kaohsiung Medical University Industry-University Cooperation Project (Grant No. S114034).

## Declaration of generative AI and AI-assisted technologies in the writing process

During the preparation of this manuscript, the authors used ChatGPT to assist with language editing and grammar checking in order to improve the clarity and readability of the text. After using these tools, the authors reviewed and edited all content as necessary.

## CRediT authorship contribution statement

**Jie-Yu Huang:** Writing – original draft. **Albright Dew Baua:** Writing – original draft. **Zih-Syuan Yang:** Data curation. **Ching-I Tsui:** Methodology. **Pin-Chen Chen:** Validation. **Wen-Hung Wang:** Writing – review & editing. **Wanchai Assavalapsakul:** Writing – review & editing. **Arunee Thitithanyanont:** Writing – review & editing. **Yen-Hsu Chen:** Writing – review & editing. **Sheng-Fan Wang:** Writing – review & editing, Supervision, Funding acquisition, Conceptualization.

## Declaration of competing interest

The authors declare that they have no known competing financial interests or personal relationships that could have appeared to influence the work reported in this paper.

## Data Availability

Data will be made available on request.
